# Policy environment for prevention, control and management of cardiovascular diseases in primary health care in Kenya

**DOI:** 10.1186/s12913-018-3152-4

**Published:** 2018-05-09

**Authors:** Gershim Asiki, Shuai Shao, Carol Wainana, Christopher Khayeka–Wandabwa, Tilahun N. Haregu, Pamela A. Juma, Shukri Mohammed, David Wambui, Enying Gong, Lijing L. Yan, Catherine Kyobutungi

**Affiliations:** 1 African Population and Health Research Center, P.O Box 10787-00100, Nairobi, Kenya; 2 Karolinska Institutet, Department of Women’s and Children’s Health, Stockholm, Sweden; 3 ACCESS Health International, Shanghai, China; 4 Global Health Research Center, Duke Kunshan University, Kunshan, China; 5 School of Pharmaceutical Science and Technology, Health Science Platform, Tianjin University, Tianjin, 300072 China; 6 Duke Global Health Institute, Duke University, Durham, NC USA

**Keywords:** Policy, Cardiovascular diseases, Prevention, management and control, Primary healthcare, Kenya

## Abstract

**Background:**

In Kenya, cardiovascular diseases (CVDs) accounted for more than 10% of total deaths and 4% of total Disability-Adjusted Life Years (DALYs) in 2015 with a steady increase over the past decade. The main objective of this paper was to review the existing policies and their content in relation to prevention, control and management of CVDs at primary health care (PHC) level in Kenya.

**Methods:**

A targeted document search in Google engine using keywords “Kenya national policy on cardiovascular diseases” and “Kenya national policy on non-communicable diseases (NCDs)” was conducted in addition to key informant interviews with Kenyan policy makers. Relevant regional and international policy documents were also included. The contents of documents identified were reviewed to assess how well they aligned with global health policies on CVD prevention, control and management. Thematic content analysis of the key informant interviews was also conducted to supplement the document reviews.

**Results:**

A total of 17 documents were reviewed and three key informants interviewed. Besides the *Tobacco Control Act (2007),* all policy documents for CVD prevention, control and management were developed after 2013. The national policies were preceded by global initiatives and guidelines and were similar in content with the global policies. The *Kenya health policy (2014–2030)*, The *Kenya Health Sector Strategic and Investment Plan (2014–2018)* and the *Kenya National Strategy for the Prevention and Control of Non-communicable diseases (2015–2020)* had strategies on NCDs including CVDs. Other policy documents for behavioral risk factors (*The Tobacco Control Act 2007, Alcoholic Drinks Control (Licensing) Regulations (2010))* were available. The *National Nutrition Action Plan (2012–2017)* was available as a draft. Although Kenya has a tiered health care system comprising primary healthcare, integration of CVD prevention and control at PHC level was not explicitly mentioned in the policy documents.

**Conclusion:**

This review revealed important gaps in the policy environment for prevention, control and management of CVDs in PHC settings in Kenya. There is need to continuously engage the ministry of health and other sectors to prioritize inclusion of CVD services in PHC.

**Electronic supplementary material:**

The online version of this article (10.1186/s12913-018-3152-4) contains supplementary material, which is available to authorized users.

## Background

Cardiovascular diseases (CVDs), account for most of the Non Communicable Disease (NCD) deaths worldwide [1]. Among all NCDs, cardiovascular diseases were responsible for 17.6 million deaths worldwide in 2015, with 75% of all CVD deaths occurring in developing countries [2]. Global initiatives such as the “Package of Essential NCD (PEN) interventions for primary care in low-resource settings”, [3, 4], a political declaration made by the United Nations high-level meeting for NCD prevention and control to strengthen primary PHC and recommendations for diagnosis and management of cancer, diabetes, heart diseases, stroke and chronic diseases for primary health care in low-resource settings in 2012 [5], were the first evidence of an increased global recognition of PHC as an important entry point for CVD prevention, control and management. Some guidelines for CVD were embedded within these general NCD strategies. In 2016, the World Health Organization (WHO) launched its first specific initiative named “*Global Hearts Initiative”* targeting CVDs at primary healthcare level. It includes three technical packages targeting tobacco epidemic, salt reduction and primary healthcare-based cardiovascular disease management [6]. The key components at primary health level proposed are summarized as **HEARTS**: **H**ealthy lifestyle (counselling on tobacco cessation, diet, physical activity and self-care), **E**vidence-based treatment protocols, **A**ccess to essential medicine and technology, **R**isk-based management, **T**eam-based care and task-sharing and **S**ystems for monitoring.

These initiatives are expected to be rolled out to several countries over time. Despite these global initiatives PHC in Sub-Saharan Africa (SSA) is still struggling to re-orient services to simultaneously respond to infectious diseases and NCDs [7]. Most countries in SSA designed their health systems with a focus on managing infectious diseases but due to the rising prevalence of NCDs, there is a need to shift to a primary healthcare approach inclusive of prevention and control of NCDs in alignment with the global initiatives. Government led response and policy changes have been achieved in some countries, however there continues to be a huge gap in the policy frameworks guiding this process in several countries in SSA.

Kenya is one of the countries in SSA experiencing a rapid demographic and epidemiological transition with a rising burden of NCDs such as CVDs, cancer, and diabetes [8]. In Kenya, CVDs alone accounted for more than 12% of total deaths and 5% of total Disability-Adjusted Life Year (DALYs) in 2016 with a steady increase over the past decade [8]. The prevention, control and management of CVDs is gaining increasing attention from policymakers. However, the policy response for adapting primary healthcare services for prevention and control of CVDs may not be in tandem with the growing burden of CVDs.

The Kenya healthcare model is a six-tiered system comprising community health services, primary healthcare (PHC) facilities (levels II and III), county hospitals, regional hospitals and national referral hospitals [9]. Community health services include all non-facility based health and related services each serving a population of about 5000 people in a community unit. PHC facility level II is first physical level of the health system that exists for every 10,000 persons on average to provide curative, preventive, or health promotion activities as outpatient services. A level III facility serves approximately 30,000 persons, allowing for at least 4 deliveries per day in addition to providing outpatient services. Level IV facilities are the primary referral facilities at county level whose services complement the primary care level to allow for a more comprehensive package close to the clients. While level V facilities within a cluster of counties provide a secondary referral services with a comprehensive set of services, together with internships, research and serving as training centers for paramedical staff. Level VI facilities refer to tertiary hospitals whose services are highly specialized and also include training of specialists, biomedical research and serve as internship and apprenticeship training centres [9]. The 2010 constitution decentralized governance to 47 county governments. Under this system, the function of primary healthcare primarily rests with county governments whilst the national government only provides policy and management of national referral hospitals.

In this paper we reviewed the existing policies and their content guiding CVD prevention, control and management in the Kenyan primary health care system to inform appropriate policy recommendations.

## Methods

This study focused mainly on a qualitative document review complemented by key informant interviews, to ascertain existing policies and their content for prevention, control and management of CVDs at the primary healthcare level in Kenya.

### Targeted document review

**Fig. 1:**
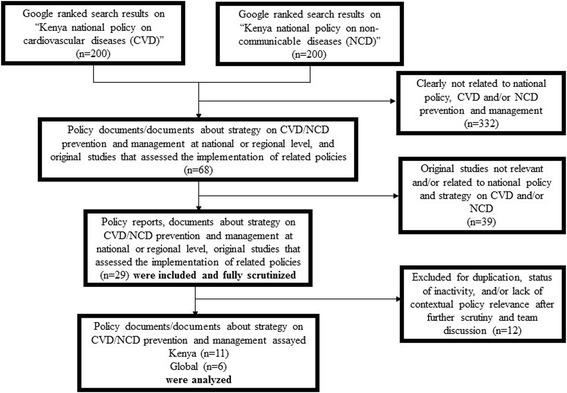
Selection process of the structured literature search of national and international Policy documents relevant to primary care based CVD control in Kenya

After consultation with local policy experts in Kenya and authors TN and CKW, a general web search was considered as the most appropriate search method in the context of Kenya. The detailed search and selection process is shown in Fig. 1. Building on the experience of prior research [10], our document search relied on the power of relevancy ranking within the Google search engine. The authors conducted two separate searches on Google search engine using keywords: “Kenya national policy on cardiovascular diseases” and “Kenya national policy on non-communicable diseases”. There were more than 257,000 results returned by Google for “Kenya national policy on cardiovascular diseases” and 701,000 results for and “Kenya national policy on non-communicable diseases”. Given it is nearly impossible to scan all retrieved results from the two separate google searches, with the guidance of prior literature [10] and the collective research team experience, the authors agreed to review the first 200 results in each Google search, to capture many of the most relevant research results while keeping the screening workload feasible for the research team.

We used prior established inclusion/exclusion criteria to narrow down the number of relevant documents. Firstly, by scanning the title and short text underneath of the combined 400 searches, the authors eliminated those search results (*n* = 332) that are clearly not related to national policy, CVD and NCD prevention and management. A second round of selection was then conducted by obtaining further information from clicking on the search result. Thirty-nine studies were further eliminated after this review and research team discussion. The remaining 29 documents were downloaded and fully scrutinized. The final stage of selection removed 12 documents that were either a duplication or inactive or deemed irrelevant after further scrutiny and team discussion.

### Policy maker interviews

We also had policymaker interviews (1 national level-Ministry of Health, Kenya, 2 county level-Nairobi county governments). The county level policy makers interviewed involved members recommended by the Nairobi city county operational research technical working group in consultation with the office of the county director of medical services. It is through the county technical working group team that we got redress to the national government ministry of health and thus the national interview was scheduled with a serving member of the recently established non-communicable disease unit within the Ministry of Health. The vertical targeted framework of identifying the policy makers interviewees through the health management and governance context (from county to national level) was considered ideal in order to reach policy makers actively mandated with formulation and implementation of policies on CVD’s and NCD prevention and management. The three participants were thus contacted by the research team via email or phone contact and the research details explained to them, were scheduled for an interview, consented and interviewed. The key informant interviews were conducted by a trained field worker at a venue and time convinient to the participants. The interviews lasted about one hour and were recorded in order to capture all information at transcription. The items in the policy marker interview guide were developed with a focus on; policy, governance, health financing, human resource, health information system, service delivery and other themes that emerged during interviews. The items in the policy marker interview guide are provided [see Additional file 1]. The county interviews were conducted in Nairobi County and the national level interviews at the ministry of health headquarters in Nairobi as part of a harmonized protocol of a broader study, implemented in four countries including China, Vietnam, Nepal and Kenya following same protocol.

### Data analysis

Qualitative document analysis was conducted using *NVivo* (QSR International’s NVivo 11 Software). Through categorizing the qualitative data into concepts and themes, evaluating the explanations and evidence, key findings on the gaps and barriers of current primary healthcare services on CVD management and prevention were identified. The content of policy documents was analyzed. Content refers to the policy goals, strategies, action plans and scientific evidence. Relevant information relating to the content was extracted from the documents into Excel sheets and analyzed.

## Results

As shown in Figs. 1, 17 documents were found to be relevant for review and analysis. These included six global policy documents and eleven national documents: policy documents, national strategies and legislation.

### Policies and content

From the documents reviewed, there was no standalone policy for CVD management and care at PHC level, but some aspects of CVD policy were covered in general NCD policy documents. The policy makers interviewed also confirmed that there was no standalone policy for CVD management and care at PHC level as in the quotation below.

*“The ministry of health and the division of non-communicable diseases has no standalone document called a policy/strategy/action plan for CVDs, what we have is national strategy for non-communicable diseases in general and CVDs are included in that strategy”.*

*“But now the roles are general for NCDs not specified for CVDs. The counties have their say in management of healthcare and so they have their guidelines and the roles defined in their management of health systems”* (Policy Maker- County).

However, there are plans underway to develop a standalone CVD policy as indicated in the quotes.

*“Currently there are two efforts that are going on. One is we are trying to develop guidelines for cardiovascular diseases because treatment of cardiovascular diseases happens haphazardly. There is no standardized way of doing things. So we want to standardize that. Secondly, we have realized that there are so many small groups that are doing pilots on NCD’s and some of them are doing on CVD. And we have realized that all those learnings have not been captured together in a document and they have not been documented well for learnings and for policy distribution. So we are in the process of trying to put all those pilots together then they have something they can tell us”* (Policy maker- National).

### The Kenya vision 2030

In the *Kenya Vision 2030* the country’s long-term development agenda is to improve the overall livelihood of Kenyans including the provision of an efficient and high quality health care system with the best standards. Revitalization of community health centers to promote preventative health care (in addition to curative interventions) were included as one of the five health-related Flagship Projects (97 in total) [11]. The direct channeling of health fund to both hospitals and community health centers was also included as a key initiative. However, the prevention, control and management of NCDs (including CVDs) was not explicitly mentioned in this document, although special attention to tuberculous, HIV/AIDS, malaria and lowering infant and maternal mortality ratio was mentioned.

### The Kenya health policy 2014–2030

In line with Article 2 of the Constitution, the Kenya *Health Policy (2014–2030)* was developed to provide long-term policy direction for health in order to reach the target of “attaining the highest possible standard of health in a responsive manner”. It recognizes the emerging trend of NCD as a key contributor to disease burden and sets specific targets to be achieved in reduction of CVD burden.

*“And in there (2014-2030 Kenya policy) were six policy directions and among the six four were directly under non-communicable diseases. This inclusion was informed by the realization that immunological transition [sic] is happening very fast in Kenya and there will be need to move away from the focus of infectious diseases…we now have it a division called the triple burden of disease and that is infectious diseases burden, non-communicable diseases and injuries”* (Policy maker, National).

*The Kenya health policy (2014–2030)* cited three objectives related to NCDs including halting and reversing the rising burden of non-communicable conditions and mental disorders, providing essential healthcare and minimizing exposure to risk factors with specific priorities target indicators (Table 1).

**Table 1:** Kenya Health Policy aspects related to prevention, control and management of CVDs at primary healthcare

Policy No	Policy Objectives	Priority Policy Strategies relating to the prevention, control and management of CVD at primary healthcare level
Policy Objective 2	Halt and reverse the rising burden of non-communicable conditions and mental disorders	Management of NCDs through a multisectoral approach to decentralize screening and surveillance, improve health service delivery, health promotion and implement targeted interventions
Policy Objective 4	Provide Essential Healthcare	NCD management through integration into Infectious disease management infrastructure, nutritional interventions, organized system for referrals and health service delivery.
Policy Objective 5	Minimize exposure to health risk factors	Mechanisms to screen and mitigate risk factors, promotion of lifestyle modification (nutrition, physical activity, controlled use of alcohol and drugs, and healthier environment), intersectoral mechanisms for regulation and promotion of healthy products and responsible marketing, facilitate and collaborate on the implementation of health research agenda

### Kenya health sector strategic and investment plan 2014–2018 (KHSSP)

Further down the hierarchy of the legislative framework is the *Kenya Health Sector Strategic and Investment Plan 2014–2018* (KHSSP), which defines the medium term focus, objectives and priorities that recognize NCDs as the leading contributor to the high burden of disease [9]. It specifically mentions health promotion for prevention of NCD, communication on harmful effect of tobacco and alcohol abuse, provision of facility based health messages on benefits and approaches to improving physical activity from Level 2 health facilities. The *Kenya Health Sector Strategic and Investment Plan 2014–2018* (KHSSP), recognizes NCDs as the leading contributor to the high burden of disease and recommend measures such as integrating health service provision tools, mechanisms and processes for responding to NCDs, establishing screening programs at community level and in health facilities for major NCDs, putting in place interventions directly addressing marginalized and indigenous populations affected by NCDs and improving working conditions, particularly in the workplaces that pre-dispose persons to NCDs. It also emphasized the role of primary health care facilities (level II) in health promotion for prevention of NCD including communication on harmful effects of tobacco and alcohol abuse, provision of facility based health messages on benefits and approaches to improving physical activity.

### The National Strategy for NCD prevention and control (2015–2020)

Finally, the *National Strategy for NCD prevention and control* (2015–2020) provides the policy direction and implementation framework CVD prevention, control and management [12]. The *Kenya National Strategy for the Prevention and Control of Non-communicable diseases 2015–2020* provides a clear policy direction and implementation framework for prevention and control of cardiovascular diseases with a focus on reducing prevalence of hypertension and diabetes and reducing behavioral risk factors such as tobacco smoking, harmful use of alcohol, physical inactivity, excessive salt consumption.

Other Specific policy documents were available for behavioral risk factors for NCDs. Kenya signed and ratified the WHO Framework Convention on Tobacco Control (WHO FCTC) in 2004 and thereafter implemented comprehensive tobacco control legislation as partial fulfillment to its FCTC obligation [13]. *The Tobacco Control Act 2007* is the principal law governing tobacco control in Kenya [14]. It aimed to regulate the packaging of tobacco products, control smoking in public places, ban direct and indirect advertising, and ban the sale of tobacco to and by minors. Kenya also passed the *Alcoholic Drinks Control (Licensing) Regulations (2010)* [15] to curb the harmful consumption of alcohol. The regulations aim to protect the general public and protect consumers of alcohol through several ways: prohibit misleading inducements to use of alcohol, limit access to alcoholic products for young people under 18 years, educate the public on the dangers of alcohol use (economic, social & health), promote and provide treatment & rehabilitation programs for those addicted and to promote research and dissemination of health risks association with alcohol consumption. The *Alcoholic Drinks Control (Amendment) Act (2015)* [16] included new imperatives such as providing support and assistance in the establishment of treatment and rehabilitation programs that shall recognize alcoholism as a disease, promoting the establishment of treatment and rehabilitation programs that are affordable, and educating the public on the benefits of using affordable alternatives to dangerous liquor.

The *National Nutrition Action Plan (2012–2017)*, provides a roadmap to implementation of nutrition interventions by the government and stakeholders, and sets out activities to scale up implementation of high-impact nutrition specific interventions which are incorporated in the health system. However, its initial focus was on food insecurity until the global strategy on diet and physical activity was passed, then healthy diets and physical activity guidelines were incorporated.

Although National NCD strategy mentioned a target of 10% relative reduction in prevalence of insufficient physical activity, no policy for physical activity was in place.

### Alignment of policies with global initiatives

The document review revealed that all policy documents related to CVD prevention, control and management were mainly driven by global initiatives and guidelines. For example, the tobacco control Act 2007, followed the Framework Convention on Tobacco Control (2003), and the Alcoholic Drinks Control (Licensing) Regulation (2010) followed the Global Strategy to reduce harmful use of Alcohol (Resolution WHA 63.13). The National Nutrition Action Plan (2012–2017) initially focused on food insecurity but after the Global Strategy on Diet, Healthy diet and Physical activity guidelines were developed. The *Global Action Plan for the Prevention and Control of NCDs 2013–2020* (the Global Action Plan) by the WHO in 2011, preceded the *National Strategy for NCD prevention and control* (2015–2020). Table 2, shows an outlines of 4 global NCD policy documents that preceded the Kenya National NCD documents and therefore informed the development of national documents.

**Table 2:** NCD policy documents in Kenya corresponding to the Global framework

Global	Kenya-specific
WHO Global Action Plan for the prevention and control of NCDs (2013–2020)	Kenya National NCD Strategy (2015)
Framework Convention on Tobacco Control, 2003	Tobacco Control Act 2007 Tobacco Control Regulation 2014 Tobacco Control Action Plan (2010–2015)
Global Strategy to reduce harmful use of Alcohol (Resolution WHA 63.13), 2010	Alcoholic Drinks Control (Licensing) Regulation (2010)
Global Strategy on Diet, Physical Activity and Health, 2004	National Nutrition Action Plan (2012–2017)

Other global initiatives that shaped the policy environment in Kenya include the *WHO NCD Global Monitoring Framework* further that laid out 25 indicators to track the mortality & morbidity, risk factors and performance of national system response as part of the comprehensive global monitoring framework for the prevention and control of NCDs [17]. More recently, the *2030 Sustainable Development Agenda* [18], *Guidelines for primary health care in low-resource settings-Cancer, diabetes, heart disease and stroke, chronic respiratory disease*, Package of Essential Non communicable diseases intervention for primary health care in low resource settings (*PEN) 2010* and *PEN 2013* are global initiatives driving the implementation of CVD prevention, management and control at PHC level [4, 5].

Table 3 Compares a selected content of the Global action plan for NCDs and the Kenya National strategy for prevention and control of NCDs and shows marked similarities in the targets further confirming that the national targets were guided by global targets.

**Table 3:** Comparison of Global and Kenya’s National policy content for selected CVD/risk factor targets

Disease Burden/Risk Factors	Global NCD Action Plan	Kenya National Strategy for the prevention and control of NCDs
	(Voluntary Global Target by 2025)	(2015–2020)
A. Disease-specific targets		
Mortality	A 25% relative reduction in risk of premature mortality from CVD, diabetes, cancer et al.	
Blood pressure	A 25% relative reduction in the prevalence of raised blood pressure	A 25% relative reduction in the prevalence of raised blood pressure
Diabetes/Obesity	0% increase in diabetes/obesity	Halt the rise in Diabetes and obesity
B. Behavioral Risk factor targets		
Tobacco Use	A 30% relative reduction in prevalence of current tobacco use in persons aged 15+ years	a. A 30% relative reduction in prevalence of current tobacco use in persons aged 15+ years
		b. A 30% relative reduction in prevalence of current tobacco use in adolescents
Excessive consumption of alcohol	At least 10% reduction in the harmful use of alcohol	At least 10% reduction in the harmful use of alcohol
Physical Inactivity	A 10% relative reduction in prevalence of insufficient physical activity	A 10% relative reduction in prevalence of insufficient physical activity
Excessive Salt Intake	A 30% relative reduction in mean population intake of salt/sodium	A 15% relative reduction in mean population intake of salt/sodium

## Discussion

This review revealed that there were no standalone policies targeting cardiovascular disease prevention, control and management at primary health level in Kenya. Most of the policy documents available were developed after 2013 and theoretically recognize the challenge of CVDs but in reality, there is no mention of integrating CVD interventions into primary health care. A primary healthcare approach is desired for prevention focused healthcare systems with the five key elements: integrated approaches including referral of care and information as required; importance of community participation; inter-sectoral focus and private-sector involvement; equity in healthcare and in health and reflecting the needs of the community; and cost-effective, evidence-based and affordable solutions, including community health workers in accordance with the Alma-Ata declaration [19, 20]. The Kenya health policy (2014–2030) and the National Strategy for NCD prevention and control (2015–2020) have highlighted some aspects of prevention, control and NCD management but with no specific reference to the role of PHC.

In light of growing burden of CVD risk factors in Kenya, there is a need to strengthen the response by the primary health care system. According to the national NCD risk factor survey conducted in 2015, 27% of adults in Kenya were either overweight or obese, nearly one quarter had hypertension [21]. The total risk of developing CVD determined by combining the effect of behavioral and biological risk factors showed that 8% of adults aged 40–69 years had a CVD risk of 30% or more with only 6% receiving drug treatment and counselling [21]. The same survey revealed that more than half of Kenyans had never measured their blood pressure and among those found with hypertension only 23% had accessed care. High sodium intake has been found to be associated with elevated risk of hypertension and cardiovascular diseases [22]. The WHO set the recommended daily intake of salt at 5 g (2 g of sodium). Kenya contributed data to a multi-country meta-analysis by the NUTRICODE study which showed that 4 CVD deaths per million adults were attributable to excessive salt consumption in Kenya [23]. Another systematic review of salt-intake in Sub-Sahara Africa in 2016 located two studies on population salt intake in Kenya conducted 1980 [24]. This abundant local evidence was expected to have guided policies for prevention, control and management of CVDs, however most existing policies were developed several years after publishing the evidence but soon after the global NCD strategies were in place. For example salt reduction was mentioned in the National NCD strategy in 2015, yet evidence of high salt intake was available in the 1980’s.

The NCD policy documents and guidelines available in Kenya have largely been guided by global initiatives as reflected in the time sequence in which Kenya policies followed global policies and the similarity in the content with some global policies. However, the WHO PEN and HEARTS global initiatives are yet to be implemented in Kenya. A review of countries progress with implementation of WHO PEN in the Africa region showed that since 2012, only nine Member States adapted WHO PEN tools in pilot districts and among them, only Benin and Togo have rolled them out nationally [25].

The policy environment at the global level is more promising as it has created an enabling environment for countries to develop their own policies. Recognizing the surging disease burdens associated with NCDs, more than 190 countries, including Kenya, pledged to the *Global Action Plan for the Prevention and Control of NCDs 2013–2020* (the Global Action Plan) by the WHO in 2011, creating an enabling environment for addressing the NCDs epidemic on a global scale [26]. Considered as an equitable and cost-effective way to achieve population health attainment, primary healthcare revitalization is a pivotal approach to address the NCD burden [27].

Indeed, there has been increasing discussion on the inclusion of the NCD prevention and control in existing primary healthcare system for other diseases [28]. This approach aims to minimize duplication of scarce resources and inputs and promote efficiency given the similarities in the underlying determinants of the major health challenges as well as the key beneficiaries of the health services.

A major limitation of this review is a small number of key informant interviews done to complement the document reviews.

## Conclusion

This review highlights important gaps in the policy environment for prevention, control and management of cardiovascular diseases in primary health care settings in Kenya. At the national level there were no standalone policies guiding CVD prevention, control and management at primary health care level, but some policy aspects were cited in the Kenya health policy (2014–2030) and Kenya National Strategy for the prevention and control of NCDs (2015). The global policy landscape raises optimism for improvement in CVD policy at primary healthcare level. Kenya’s devolved system of governance to counties offers both challenges and opportunities to strengthen the primary healthcare system at the grassroots. There is need to continuously engage the ministry of health and other sectors to embrace a primary healthcare approach which integrates CVD prevention, management and control.

## Additional file

Additional file 1: Policy marker interview guide for key informant interviews. Description of data: Sample of interview guide for key informant interviews with policy makers. (DOCX 15 kb)

## Abbreviations

CVDs: Cardiovascular diseases, 4
DALYs: Disability-Adjusted Life Year,4
HEARTS: Healthy lifestyle (counselling on tobacco cessation, diet, physical activity and self-care), Evidence-based treatment protocols, Access to essential medicine and technology, Risk-based management, Team-based care and task-sharing and Systems for monitoring., 4
KHSSP: *Kenya Health Sector Strategic and Investment Plan 2014-2018*, 13
NCD: Non Communicable Disease, 4
NHIF: National Health Insurance Fund, 8
PEN: Package of Essential Non communicable diseases intervention for primary health care in low resource settings, 11
PHC: Primary health care, 4
SAA: Sub-Saharan Africa, 4
VAT: value-added tax, 20
WHO FTC: WHO Framework Convention on Tobacco Control, 14
WHO: World Health Organization (WHO), 4
