# Supplementary material for: Policy environment for prevention, control and management of cardiovascular diseases in primary health care in Kenya
Source: BMC Health Serv Res. 2018 May 9;18:344. doi: 10.1186/s12913-018-3152-4 (PMC5944159; doi:10.1186/s12913-018-3152-4)
Supplement: Supplementary file 1 — Policy marker interview guide for key informant interviews. Description of data: Sample of interview guide for key informant interviews with policy makers. (DOCX 15 kb) [file 12913_2018_3152_MOESM1_ESM.docx]

**Policy maker interview guide**

1. **Policy**

• Are there national policy/ strategy/ action plan for CVDs?

• Are the roles and responsibilities of PHC highlighted in the policy? Specify if any.

1. **Governance**

• Which departments/ units/ groups are responsible for planning, implementing, supervising and managing CVDs related interventions? What are their roles and responsibilities? Are there any collaboration between multiple departments?

o Departments/units/groups include:

♣ Health sector (at central, provincial, district and commune levels)

♣ Other public sectors (finance, education, trade, etc…)?

♣ Private sector

♣ Community organizations (women union, youth union, famer unions, etc) ?

• Is there any guideline available in the primary healthcare level in terms of CVD patients (especially hypertensive patients) management and prevention? Can you show us the guideline?

1. **Health Financing**

• Who are responsible for financing CVD related interventions implemented at PHC level (state budget, health insurance, foreign aids, out-of-pocket payment, others)? What is the trend of changes overtime?

• Role of state budget: From central and local government? What cost items are covered by the state budget? How is the budget estimated? How is the budget allocated?

• Role of health Insurance: Provider payment mechanism?

• Role of foreign aids: Sustainability?

• Role of out-of-pocket payment: How user fee is set and collected? How the facilities utilize the fee collected? Regulation to control irrational care?

1. **Human resources**

• What do you think of the current situation of CVD management and prevention in terms of human resources in primary healthcare level? Is there enough specialist working on this area? Is the training to health providers in terms of CVD management and prevention enough?

1. **Health information system**

• What is the current health information system in primary healthcare level? Do you think the current health information system in primary healthcare level is enough for prevent and manage CVD patients? What do you think is the priority in terms of improving health information system in primary healthcare level?

• What do you think about the feasibility of future implementation of M-health to help prevent and manage CVDs? What are the possible barriers?

1. **Service Delivery**

• What services about CVD are available at primary healthcare level (population based health promotion program/ early detection of people at high risks/ early treatment of high risk patients/ rehabilitative care)?

• Is there any issues related to access, utilization, quality, equity?

1. **Others**

• What do you think about the current situation of primary healthcare in terms of providing CVD prevention and management to general population?

• What do you think about the future of primary healthcare in terms of providing CVD prevention and management to general population?
